# A Fractional-Order Sinusoidal Discrete Map

**DOI:** 10.3390/e24030320

**Published:** 2022-02-23

**Authors:** Xiaojun Liu, Dafeng Tang, Ling Hong

**Affiliations:** 1 School of Sciences, Xi’an University of Posts and Telecommunications, Xi’an 710061, China; 2 School of Automation, Xi’an University of Posts and Telecommunications, Xi’an 710061, China; tdflyy2011@163.com; 3 State Key Laboratory for Strength and Vibration of Mechanical Structures, Xi’an Jiaotong University, Xi’an 710049, China; hongling@mail.xjtu.edu.cn

**Keywords:** a fractional-order discrete map, chaos, bifurcation, synchronization

## Abstract

In this paper, a novel fractional-order discrete map with a sinusoidal function possessing typical nonlinear features, including chaos and bifurcations, is proposed. Firstly, the basic properties involving the stability of the equilibrium points and the symmetry of the map are studied by theoretical analysis. Secondly, the dynamics of the map in commensurate-order and incommensurate-order cases with initial conditions belonging to different basins of attraction is investigated by numerical simulations. The bifurcation types and influential parameters of the map are analyzed via nonlinear tools. Hopf, period-doubling, and symmetry-breaking bifurcations are observed when a parameter or an order is varied. Bifurcation diagrams and maximum Lyapunov exponent spectrums, with both a variation in a system parameter and an order or two orders, are shown in a three-dimensional space. A comparison of the bifurcations in fractional-order and integral-order cases shows that the variation in an order has no effect on the symmetry-breaking bifurcation point. Finally, the heterogeneous hybrid synchronization of the map is realized by designing suitable controllers. It is worth noting that the increase in a derivative order can promote the synchronization speed for the fractional-order discrete map.

## 1. Introduction

In the last few years, the study of discrete chaotic systems has been a point of discussion in the fields of control and secure communication. Two principal reasons for this attention are the chaotic nature and the discrete nature of these kinds of systems. The chaotic nature seems random but is, indeed, completely determined and can be predicted when the initial conditions are known. The discrete nature allows for simple implementation and reduced computational complexity. Therefore, many typical discrete chaotic maps are presented, such as the Logistic map, the Hénon map, and the Lozi map [1,2,3,4,5].

It is well known that fractional calculus plays a crucial role in many areas, such as electric fields, population inversion, electromagnetic fields, and secure communication [6,7,8,9,10]. In 1989, Miller and Ross first introduced the υ order fractional sum and the fractional integral as a fractional sum [11]. Indeed, the first fractional-order maps were derived from fractional differential equations [12]. The new dynamical properties of the fractional-order dynamical systems were revealed [13]. Due to further research, more attention has been paid to the fractional discrete chaotic systems which involve the discrete fractional calculus [14,15,16,17]. Compared with the continuous fractional calculus, the discrete ones can avoid the tedious information and calculation error of the numerical discretization result on account of the non-local property of the operator [18]. Such dynamical systems described by fractional difference equations are related to several areas, including viscoelasticity, electrochemistry, diffusion processes, automatic control, and power electronics [19,20,21,22,23,24,25].

A discrete chaotic map involving fractional calculus has complex dynamics. Furthermore, it is not only sensitive to a small disturbance in parameters and initial conditions, but also to the change in fractional orders [26]. Therefore, fractional-order discrete maps, with simple forms and rich dynamics, are more suitable for data encryption and secure communication [27,28,29]. To this end, the study of a new fractional-order discrete map is necessary and important for the development of fractional calculus and dynamics. Recent reports discuss subjects including: the novel convenient condition for the stability of fractional-order difference systems in the incommensurate-order case [30]; the complex dynamics in the discrete memristor-based system with fractional-order difference [31]; the chaos and projective synchronization of a fractional-order difference map with no equilibria [32]; and the rich dynamical characteristics of a new fractional-order, 2D discrete chaotic map [33]. These works mainly focus on the stability, dynamics, bifurcation, and synchronization of fractional-order discrete maps. Moreover, the multistability and coexisting bifurcation phenomena also exist in fractional chaotic maps [34,35,36] which have many applications in chaotic-based engineering. The maps with the characteristic of multistability change the steady state of small disturbances on the initial conditions. Therefore, determining the steady state of a dynamical system with a certain condition is a challenge to the theoretical analysis and the numerical simulation. However, there are very few reports about the effect of the derivative order on the symmetry-breaking bifurcation point and synchronization speed for a fractional-order discrete map.

In [37,38], a new two-dimensional sinusoidal discrete map is proposed by nonlinearly coupling a sinusoidal map with a cubic map. The research results show that the map possesses complex dynamics, including chaos, symmetry-breaking, and Hopf bifurcations. Based on these results, we want to know whether these complex dynamics still exist in the corresponding fractional mode of the map. We know that the order is a very important parameter for a fractional-order system, which is remarkably different from an integral-order system. The effect of the order on the dynamics is necessary for the development and the application of fractional calculus.

Inspired by the aforementioned research background, this paper presents a novel fractional-order discrete map with a sinusoidal function possessing typical nonlinear features, including chaos and bifurcations. The basic properties of the map, such as its stability and symmetry, are studied based on theoretical analysis. The bifurcation types and influential parameters for the map are investigated via nonlinear tools. The heterogeneous hybrid synchronization of the map is realized.

## 2. Discrete Fractional Calculus

In this section, the definitions and theories relating to discrete fractional calculus will be recalled. In the rest of the paper, the symbol ${}^{C}\Delta_{a}^{\upsilon }X(t)$ means the υ order fractional calculus in the sense of Caputo type delta for a function $X(t):N_{a}\to R$ with $N_{a}=\{a,a+1,a+2,\cdots \}$ [39]. This can be described as follows:

(1) $${}^{C}\Delta_{a}^{\upsilon }X(t)={\Delta_{a}}^{-(n-\upsilon )}\Delta^{n}X(t)=\frac{1}{\Gamma (n-\upsilon )}\sum_{s=a}^{t-(n-\upsilon )}{\left(t-s-1\right)}^{\left(n-\upsilon -1\right)}\Delta_{s}^{n}X(s),$$

where υ∉N represents the derivative order, $t\in N_{a+n-\upsilon }$, and $n=\left\lceil \upsilon \right\rceil +1$, the υ fractional sum of $\Delta_{s}^{n}X(t)$ in (1) is defined as

(2) $${\Delta_{a}}^{-\upsilon }X(t)=\frac{1}{\Gamma (\upsilon )}\sum_{s=a}^{t-\upsilon }{\left(t-s-1\right)}^{\left(\upsilon -1\right)}X(s)$$

where $t\in N_{a+\upsilon }$ and υ>0 [40]. The symbol $t^{\left(\upsilon \right)}$ means the falling function, which can be denoted according to the Gamma function Γ as

(3) $$t^{\left(\upsilon \right)}=\frac{\Gamma (t+1)}{\Gamma (t+1-\upsilon )}$$

The numerical solutions for a fractional-order discrete map can be obtained via the following method. For a fractional difference equation [41]

(4) $$\left\{\begin{array}{l} {}^{C}\Delta_{a}^{\upsilon }x(t)=f(t+\upsilon -1,x(t+\upsilon -1)),\\ \Delta^{k}x(a)=x_{k}.n=\left\lceil \upsilon \right\rceil +1,\;k=0,1,2,\cdots ,n-1, \end{array}\right.$$

we can obtain the equivalent discrete integral one

(5) $$x(t)=x_{0}(t)+\frac{1}{\Gamma (\upsilon )}\sum_{s=a+n-\upsilon }^{t-\upsilon }{\left(t-s-1\right)}^{\left(\upsilon -1\right)}f(s+\upsilon -1,x(s+\upsilon -1)),\;t\in N_{a+n},$$

where $x_{0}(t)=\sum_{k=0}^{n-1}\frac{{\left(t-a\right)}^{\left(k\right)}}{\Gamma (k+1)}\Delta^{k}x(a)$.

The following theorem is frequently used to estimate the stability of a zero equilibrium point for a fractional discrete map. For the proof of the theorem, please refer to the literature [42].

**Theorem** **1.** *The zero equilibrium of a linear fractional discrete system:*

(6) $${}^{C}\Delta_{a}^{\upsilon }X(t)=MX(t+\upsilon -1),$$

*here*$X(t)={\left(x_{1}(t),x_{2}(t),\cdots ,x_{n}(t)\right)}^{T},\;0<\upsilon \leq 1,\;M\in R^{n\times n}\;\mathrm{and}\;\forall t\in N_{a+1-\upsilon }$*, is asymptotically stable if*

(7) $$\left|\lambda_{i}\right|<{\left(2\cos\frac{\left|\arg\lambda_{i}\right|-\pi }{2-\upsilon }\right)}^{\upsilon }\;\mathrm{and}\;\left|\arg\lambda_{i}\right|>\frac{\upsilon \pi }{2},i=1,2,\cdots ,n$$

For all the eigenvalues λ of M.

Here, we will give the definitions of commensurate-order and incommensurate-order fractional-order systems.

**Definition** **1.** *For a fractional-order system, which can be described by*${}^{C}\Delta_{a}^{\upsilon }=f(x(t))$*, where*$x={\left(x_{1},x_{2},\cdots ,x_{n}\right)}^{T}$*is the state vector,*$\upsilon ={\left(\upsilon_{1},\upsilon_{2},\cdots ,\upsilon_{n}\right)}^{T}$*is the fractional derivative orders vector, and*$\upsilon_{i}>0$*. The fractional-order system is a commensurate-order system when all the derivative orders satisfy*$\upsilon_{1}=\upsilon_{2}=\cdots =\upsilon_{n}$*; otherwise, it is an incommensurate-order system* [43].

## 3. A Fractional-Order Discrete Sinusoidal Map

### 3.1. Description of the Map

The two-dimensional discrete map proposed in [37,38] can be described by the following equations:

(8) $$\left\{\begin{array}{l} x(n+1)=\sin(\pi y(n)),\\ y(n+1)=c(1-x^{2}(n))y(n), \end{array}\right.$$

where x(n), y(n) are the state variables and c is a parameter. We can easily determine the first-order difference of (8), which is formulated as

(9) $$\left\{\begin{array}{l} \Delta x(n)=x(n+1)-x(n)=\sin(\pi y(n))-x(n),\\ \Delta y(n)=y(n+1)-y(n)=c(1-x^{2}(n))y(n)-y(n). \end{array}\right.$$

The corresponding fractional-order discrete map is

(10) $$\left\{\begin{array}{l} {}^{C}\Delta_{a}^{\upsilon }x(t)=\sin(\pi y(t-1+\upsilon ))-x(t-1+\upsilon ),\\ {}^{C}\Delta_{a}^{\upsilon }y(t)=c(1-x^{2}(t-1+\upsilon ))y(t-1+\upsilon )-y(t-1+\upsilon ), \end{array}\right.$$

which is determined by using the Caputo-like delta difference with the starting point a. Based on Equations (4) and (5), we can obtain

(11) $$\left\{\begin{array}{c} x(t)=x(a)+\frac{1}{\Gamma (\upsilon )}\sum_{s=a+1}^{t-\upsilon }{\left(t-s-1\right)}^{\left(\upsilon -1\right)}(\sin(\pi y(t-1+\upsilon ))-x(t-1+\upsilon )),\\ y(t)=y(a)+\frac{1}{\Gamma (\upsilon )}\sum_{s=a+1}^{t-\upsilon }{\left(t-s-1\right)}^{\left(\upsilon -1\right)}(c(1-x^{2}(t-1+\upsilon ))y(t-1+\upsilon )-y(t-1+\upsilon )), \end{array}\right.$$

where $\frac{{\left(t-s-1\right)}^{\left(\upsilon -1\right)}}{\Gamma (\upsilon )}$ means the discrete kernel function, and $\frac{{\left(t-s-1\right)}^{\left(\upsilon -1\right)}}{\Gamma (\upsilon )}=\frac{\Gamma (t-s)}{\Gamma (\upsilon )\Gamma (t-s-\upsilon +1)}$. Therefore, the numerical solution of (10) is

(12) $$\left\{\begin{array}{l} x(n)=x(a)+\frac{1}{\Gamma (\upsilon )}\sum_{j=1}^{n}\frac{\Gamma (n-j+\upsilon )}{\Gamma (n-j+1)}(\sin(\pi y(j-1))-x(j-1)),\\ y(n)=y(a)+\frac{1}{\Gamma (\upsilon )}\sum_{j=1}^{n}\frac{\Gamma (n-j+\upsilon )}{\Gamma (n-j+1)}(c(1-x^{2}(j-1))y(j-1)-y(j-1)). \end{array}\right.$$

In this paper, the low limit a is fixed as 0.

### 3.2. Symmetry and Stability of Equilibrium Points

The fractional-order Map (10) is symmetric because the transformation S:(x,y)→(−x,−y) holds, which permits the map invariant for all values of the parameters with the transformation. For this reason, all attractors of the map will appear in mutually symmetric pairs. This exact symmetry represents an important feature which demonstrates the occurrence of multiple co-existing stable states in the state space [44].

In the following, the stability of the equilibrium points of the map will be studied. Through simple computation, we can cause Map (10) to have only one equilibrium point $E_{1}(0,0)$ when c≤1, and two equilibrium points $E_{2,3}(\pm \sqrt{1-1/c},\pm \frac{1}{\pi }\arcsin\sqrt{1-1/c})$ when c>1. $E_{2}$ and $E_{3}$ are symmetric with respect to the origin $E_{1}$ and, thus, share the same stability property. The Jacobian matrix of the map evaluated at any equilibrium point $E_{*}=(x^{*},y^{*})$ is computed as follows:

$$J_{1}=\left[\begin{array}{cc} -1 & \pi \cos\pi y^{*}\\ -2cx^{*}y^{*} & c(1-x^{*2})-1 \end{array}\right]$$

The eigenvalues corresponding to the equilibrium point $E_{1}(0,0)$ are $\lambda_{1}=-1,\;\lambda_{2}=c-1$. Only the case of Map (10) with real parameters is considered in this paper. According to Theorem 1, the zero equilibrium point $E_{1}$ is unstable due to $\left|\arg\lambda_{1}\right|=0<\frac{\upsilon \pi }{2}$.

For a zero equilibrium point of fractional-order discrete maps, the stability can be determined based on Theorem 1. For a non-zero equilibrium point, a very simple method presented in [18] can be used to handle it. For further details about the method, please refer to Remark 2.5 in the literature [18].

To study the stability of the non-zero equilibrium points $E_{2,3}$, we let $x_{2}=\sqrt{1-\frac{1}{c}},y_{2}=\frac{1}{\pi }\arcsin\sqrt{1-\frac{1}{c}},x_{3}=-\sqrt{1-\frac{1}{c}},y_{3}=-\frac{1}{\pi }\arcsin\sqrt{1-\frac{1}{c}}$, and introduce the following variables, transforming

$$\left\{\begin{array}{l} z_{21}(t-1+\upsilon )=x(t-1+\upsilon )-x_{2},z_{22}(t-1+\upsilon )=y(t-1+\upsilon )-y_{2},\\ z_{31}(t-1+\upsilon )=x(t-1+\upsilon )-x_{3},z_{32}(t-1+\upsilon )=y(t-1+\upsilon )-y_{3}. \end{array}\right.$$

Two new maps with zero equilibrium points are obtained:

(13) $$\left\{\begin{array}{c} {}^{C}\Delta_{a}^{\upsilon }(z_{21}(t)+x_{2})={}^{C}\Delta_{a}^{\upsilon }z_{21}(t)=\sin(\pi z_{22}(t-1+\upsilon )+y_{2})-z_{21}(t-1+\upsilon )-x_{2},\\ {}^{C}\Delta_{a}^{\upsilon }(z_{22}(t)+y_{2})={}^{C}\Delta_{a}^{\upsilon }z_{22}(t)=c(1-{\left(z_{21}(t-1+\upsilon )+x_{2}\right)}^{2})(z_{22}(t-1+\upsilon )+y_{2})-z_{22}(t-1+\upsilon )-y_{2}, \end{array}\right.$$

and

(14) $$\left\{\begin{array}{c} {}^{C}\Delta_{a}^{\upsilon }(z_{31}(t)+x_{3})={}^{C}\Delta_{a}^{\upsilon }z_{31}(t)=\sin(\pi z_{32}(t-1+\upsilon )+y_{3})-z_{31}(t-1+\upsilon )-x_{3},\\ {}^{C}\Delta_{a}^{\upsilon }(z_{32}(t)+y_{3})={}^{C}\Delta_{a}^{\upsilon }z_{32}(t)=c(1-{\left(z_{31}(t-1+\upsilon )+x_{3}\right)}^{2})(z_{32}(t-1+\upsilon )+y_{3})-z_{32}(t-1+\upsilon )-y_{3}, \end{array}\right.$$

which correspond to $E_{2,3}$, respectively. For Maps (13) and (14), the Jacobian matrixes evaluated at the zero equilibrium point are

$$J_{2}=\left[\begin{array}{cc} -1 & \pi \cos(\pi y_{2})\\ -2cx_{2}y_{2} & c(1-{x_{2}}^{2})-1 \end{array}\right]\;and\;J_{3}=\left[\begin{array}{cc} -1 & \pi \cos(\pi y_{3})\\ -2cx_{3}y_{3} & c(1-{x_{3}}^{2})-1 \end{array}\right]$$

By simple calculation, we can obtain the eigenvalues of $J_{2}$

$$\lambda_{3,4}=\frac{c}{2}\pm \frac{\sqrt{c({cx_{2}}^{4}-2c{x_{2}}^{2}-8\pi x_{2}y_{2}\cos(\pi y_{2}))+c}-{cx_{2}}^{2}-2}{2},$$

and the eigenvalues of $J_{3}$

$$\lambda_{5,6}=\frac{c}{2}\pm \frac{\sqrt{c({cx_{3}}^{4}-2c{x_{3}}^{2}-8\pi x_{3}y_{3}\cos(\pi y_{3}))+c}-{cx_{3}}^{2}-2}{2}.$$

From the above results, we can see that the stability of the equilibrium points strongly depends on the parameter c. If c=2.5 and υ=0.98, the eigenvalues are $\lambda_{3,5}=-0.5+1.3858i$, and $\lambda_{4,6}=-0.5-1.3858i$. Based on Theorem 1, we can obtain

$$\left|\arg\lambda_{i}\right|=1.9171>\frac{\upsilon \pi }{2}=1.5379,\;i=3,4,5,6,$$

$$\left|\lambda_{i}\right|=2.0736>{\left(2\cos\frac{\left|\arg\lambda_{i}\right|-\pi }{2-\upsilon }\right)}^{\upsilon }=0.7285,\;i=3,4,5,6,\;$$

which implies that the equilibrium points $E_{2,3}$ are unstable.

## 4. Dynamics of the Fractional-Order Discrete Map

### 4.1. The Commensurate-Order Case

In this subsection, dynamics of Map (10) in commensurate-order case with different parameters and initial conditions will be studied.

**Figure 1:**
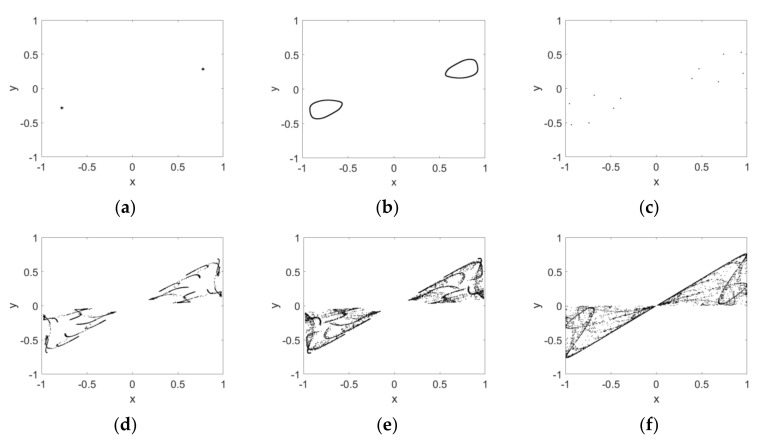
Phase diagram of the map as the order increases from 0.5 to 0.99 with IN1 and IN2. (**a**) υ=0.5; (**b**) υ=0.7; (**c**) υ=0.8; (**d**) υ=0.89; (**e**) υ=0.9; (**f**) υ=0.99.

**Figure 2:**
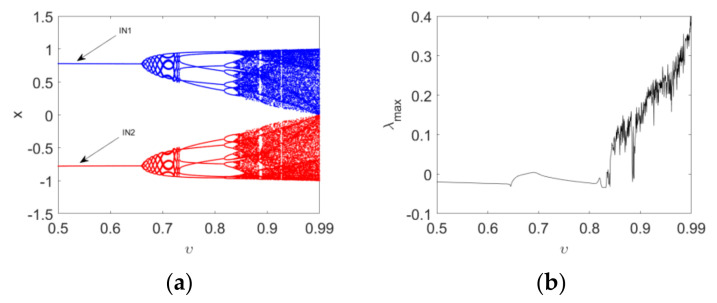
Bifurcation diagrams and maximum Lyapunov spectrum of Map (10) with the variation in the order υ when c=2.5: (**a**) the bifurcation diagrams with IN1 and IN2; (**b**) the corresponding maximum Lyapunov spectrum. Blue and red diagrams are produced by scanning the order downwards starting with IN1 and IN2.

Firstly, the parameter c is fixed as 2.5 and two initial conditions are taken as IN1=(0.2,0.2) and IN2=(−0.2,−0.2); the attractors of the map are depicted in Figure 1 as the derivative order υ varies. The intervals x∈[−1,1] and y∈[−1,1] are taken as the reference region. From this, it can be seen that the map has two mutually symmetric fixed points in the reference region when υ=0.5. The two points follow a Hopf bifurcation and give rise to a pair of limit cycles when υ increases to 0.7; this can be seen in Figure 1a,b. The map has two symmetric six-period attractors for υ=0.8, evident in Figure 1c. As the order increases further to 0.99, the route to chaos of the map is the period-doubling bifurcation. Furthermore, we can see that the two symmetric single-scroll chaotic attractors approach each other gradually in the reference region (Figure 1d,f). It is worth pointing out that the merger of two attractors, however, cannot be observed even though υ=0.99. The bifurcation diagrams and the corresponding maximum Lyapunov exponent spectrum, with respect to υ, are depicted in Figure 2. Each bifurcation diagram in the figure shows plots of local maxima of the map coordinate x in terms of υ, where blue and red diagrams are produced with the initial conditions IN1 and IN2, respectively. Hopf and period-doubling bifurcations can also be verified by Figure 2a. The maximum Lyapunov exponent spectrums, which represent the qualitative properties of dynamics, coincide with each other for IN1 and IN2, as shown in Figure 2b. It clearly shows the change from period to chaos of Map (10) as the order varies.

**Figure 3:**
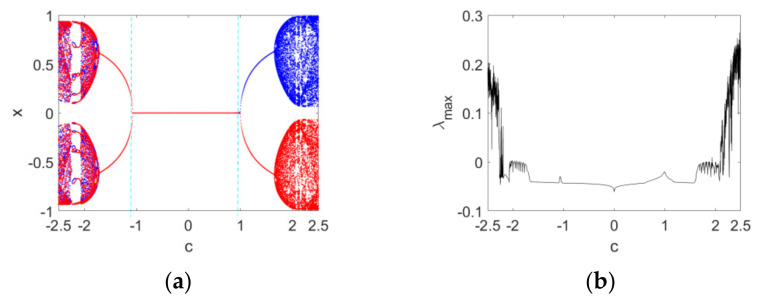
Bifurcation diagrams and maximum Lyapunov spectrum of Map (10) as the parameter c varies when υ=0.95: (**a**) the bifurcation diagrams with IN1 and IN2; (**b**) the corresponding maximum Lyapunov spectrum. Blue and red diagrams are produced with the initial conditions IN1 and IN2, respectively.

Secondly, the order υ=0.95 is fixed and the bifurcation diagrams and maximum Lyapunov exponent spectrum versus c with IN1 and IN2 are plotted in Figure 3. From Figure 3a we can see that a positive solution branch (blue) in the bifurcation diagram is corresponding to the positive initial condition IN1, while a negative solution branch (red) is corresponding to the negative initial condition IN2. The map stabilizes at the equilibrium point $E_{1}$ when −1≤c<1. A typical symmetry-breaking bifurcation occurs when c=1. The maximum Lyapunov exponent spectrum in Figure 3b, showing certain chaotic and periodic features, is consistent with the bifurcation diagrams in Figure 3a. Here, the maximum Lyapunov exponent is computed based on the Jacobian matrix algorithm for discrete fractional maps [45].

**Figure 4:**
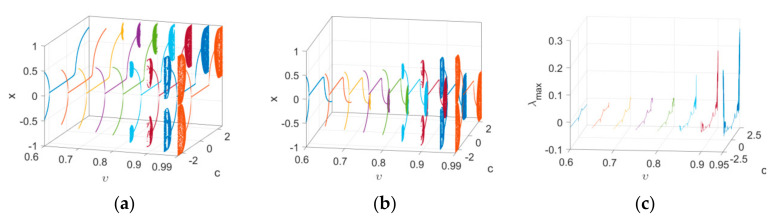
Bifurcation diagrams and maximum Lyapunov exponent spectrums in a three-dimensional space with different initial values as the parameter c and the order υ vary: (**a**) the bifurcation diagram with IN1; (**b**) the bifurcation diagram with IN2; (**c**) the corresponding maximum Lyapunov exponent spectrums.

Thirdly, the dynamics of the map with the variation in both c and υ is studied. The change in the range of c is −2.5≤c≤2.5, and that of the order υ is 0.6≤υ≤0.99. The corresponding bifurcation diagrams and maximum Lyapunov exponent spectrums in three-dimensional space are depicted in Figure 4 with IN1 and IN2. From Figure 4a,b, it can be seen that the dynamics of Map (10) with the variation in c becomes regular as υ decreases to 0.6, and complex as υ increases to 0.99. The qualitative behavior of the map can be reflected by the maximum Lyapunov exponent spectrums in Figure 4c.

**Figure 5:**
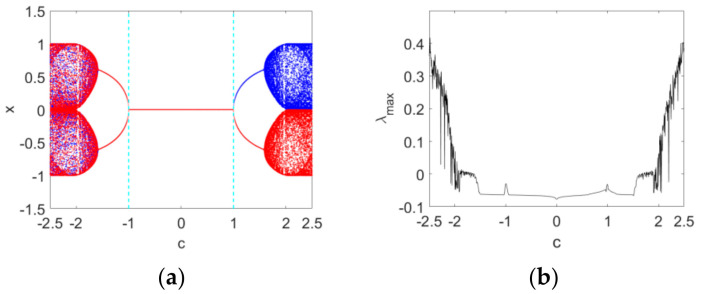
Bifurcation diagrams and maximum Lyapunov spectrum of the Map (10) as the parameter c varies when υ=1: (**a**) the bifurcation diagrams with the IN1 and IN2; (**b**) the corresponding maximum Lyapunov exponent spectrum.

Finally, in this case, we focus on the dynamics of the map with integral-order. The bifurcation diagram of the map versus the parameter c with IN1 and IN2 is shown in Figure 5a. The corresponding maximum Lyapunov exponent spectrum is depicted in Figure 5b. Thus, it is evident that the qualitative properties of the dynamics of the map is similar to the case of the fractional-order one. The typical symmetric-breaking bifurcation can also be observed when c=1, which means that the increase in the order does not affect the bifurcation point of the symmetric-breaking bifurcation. Comparing Figure 3a with Figure 5a, we can see that the notable difference is that the bifurcation point of the map from period-2 to the fixed point $E_{1}$ is slightly greater than c=−1 when υ<1, and equal to c=−1 when υ=1. In this case, it implies that the order affects the bifurcation point. Therefore, the results demonstrate that the order is a very important bifurcation parameter which affects the dynamics of the map.

### 4.2. The Incommensurate-Order Case

In this subsection, the dynamics of Map (10) in incommensurate-order case with different parameters and initial conditions will be investigated. The incommensurate-order case of Map (10) can be written in the following form:

(15) $$\left\{\begin{array}{l} {}^{C}\Delta_{a}^{\upsilon_{1}}x(t)=\sin(\pi y(t-1+\upsilon_{1}))-x(t-1+\upsilon_{1}),\\ {}^{C}\Delta_{a}^{\upsilon_{2}}y(t)=c(1-x^{2}(t-1+\upsilon_{2}))y(t-1+\upsilon_{2})-y(t-1+\upsilon_{2}), \end{array}\right.$$

where $\upsilon_{1}$ and $\upsilon_{2}$ denote the derivative orders.

**Figure 6:**
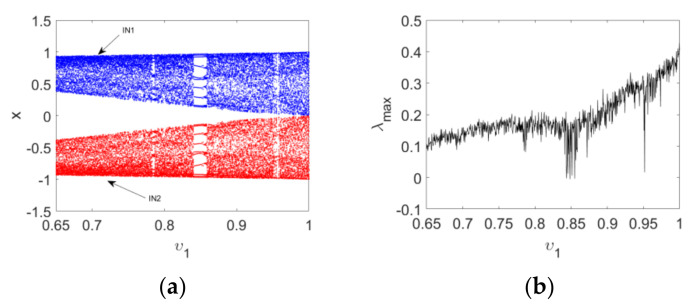
Bifurcation diagrams and maximum Lyapunov spectrum of Map (15) as the order $\upsilon_{1}$ varies: (**a**) the bifurcation diagrams with IN1 and IN2; (**b**) the corresponding maximum Lyapunov spectrum.

**Figure 7:**
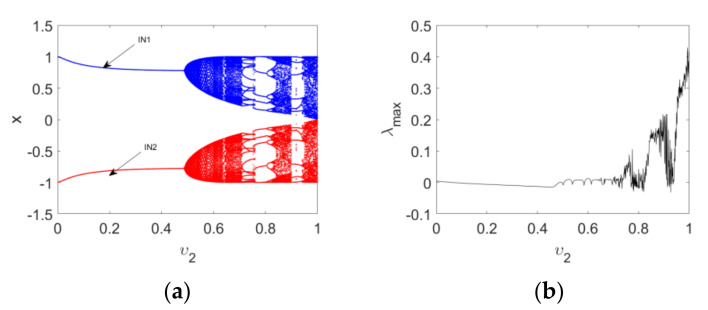
Bifurcation diagrams and maximum Lyapunov spectrum of Map (15) as the order $\upsilon_{2}$ varies: (**a**) the bifurcation diagrams with IN1 and IN2; (**b**) the corresponding maximum Lyapunov spectrum.

**Figure 8:**
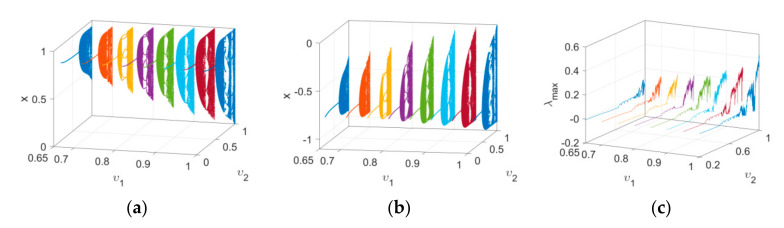
Bifurcation diagrams and maximum Lyapunov exponent spectrums in a three-dimensional space with different initial values as $\upsilon_{1}$ and $\upsilon_{2}$ vary: (**a**) the bifurcation diagram with IN1; (**b**) the bifurcation diagram with IN2; (**c**) the corresponding maximum Lyapunov exponent spectrums.

Firstly, the bifurcation and maximum Lyapunov exponent spectrum of Map (15) versus $\upsilon_{1}$ with IN1 and IN2 when $\upsilon_{2}=1$ and c=2.5 are plotted in Figure 6, which reflects the effect of order $\upsilon_{1}$ on the dynamics of the map. It is clear that periodic and chaotic windows appear alternately with the variation in $\upsilon_{1}$. Secondly, the bifurcation and corresponding maximum Lyapunov exponent spectrum of Map (15) versus $\upsilon_{2}$ are depicted in Figure 7. It can be observed that the route to chaos of the map is a typical Hopf bifurcation. The period-doubling bifurcations and chaotic windows appear alternately with the variation in $\upsilon_{2}$. Finally, the change intervals of $\upsilon_{1}$ and $\upsilon_{2}$ are set as $0.65\leq \upsilon_{1}\leq 1$ and $0\leq \upsilon_{2}\leq 1$, respectively. Bifurcations of Map (15), with the variation of two orders, are shown in a three-dimensional space, evident in Figure 8a,b. It can be observed from the maximum Lyapunov exponent spectrums in Figure 8c that the chaotic region becomes larger and chaos intensity strengthens.

Through a comparison of the map with those in [31,32,33,34,35,36], we can see that it has a typical symmetry, which may cause the symmetry-breaking bifurcation as a parameter varies. Meanwhile, the coexisting attractors also exist in the fractional chaotic map. In the aspect of algorithms, the bifurcation diagrams and the maximum Lyapunov exponent spectrums, in a three-dimensional space, give a clear presentation of the dynamics with the variation in a parameter and an order.

## 5. Heterogeneous Hybrid Synchronization

In this section, the heterogeneous hybrid synchronization of Map (10) will be investigated.

A fractional discrete Lorenz map studied in [46] is taken as the drive system, which is given by the following fractional equations:

(16) $$\left\{\begin{array}{l} {}^{C}\Delta_{a}^{\upsilon }x_{1}(t)=\gamma \delta x_{1}(\omega )-\delta y_{1}(\omega )x_{1}(\omega ),\\ {}^{C}\Delta_{a}^{\upsilon }y_{1}(t)=\delta (-y_{1}(\omega )+x_{1}{}^{2}(\omega )), \end{array}\right.$$

here 0<υ<1. The Map (16) has a chaotic attractor when γ=1.25, δ=0.75 and υ=0.98. The controlled Map (10) is as follows:

(17) $$\left\{\begin{array}{l} {}^{C}\Delta_{a}^{\upsilon }x_{2}(t)=\sin(\pi y_{2}(\omega ))-x_{2}(\omega )+u_{1}(\omega ),\\ {}^{C}\Delta_{a}^{\upsilon }y_{2}(t)=c(1-x_{2}{}^{2}(\omega ))y_{2}(\omega )-y_{2}(\omega )+u_{2}(\omega ), \end{array}\right.$$

where $u_{1}(\omega )$ and $u_{2}(\omega )$ are the hybrid synchronization controllers. The error state variables are defined as $e_{1}(t)=x_{2}(t)-x_{1}(t),\;e_{y}(t)=y_{2}(t)+y_{2}(t)$. If the two error state variables tend to zero as t→∞, Maps (16) and (17) are synchronized. It should be mentioned that $e_{1}(t)\to 0$ means the state variables $x_{1}(t)$ and $x_{2}(t)$ are synchronized, and $e_{2}(t)\to 0$ means state variables $y_{1}(t)$ and $y_{2}(t)$ are anti-synchronized. Therefore, the mode of synchronization of Maps (16) and (17) is hybrid.

The following theorem is given to ensure that the synchronization between the two maps can be realized.

**Theorem** **2.** *The two Maps (16) and (17) are synchronized if the controllers are designed as follows:*

(18) $$\left\{\begin{array}{l} u_{1}(\omega )=x_{1}(\omega )-\sin(\pi y_{2}(\omega ))+\gamma \delta x_{1}(\omega )-\delta y_{1}(\omega )x_{1}(\omega ),\\ u_{2}(\omega )=-c(1-x_{2}{}^{2}(\omega ))y_{2}(\omega )-\delta (-y_{1}(\omega )+x_{1}{}^{2}(\omega ))-y_{1}(\omega ). \end{array}\right.$$

**Proof.**  By simple calculation, we can obtain the error dynamical system

(19) $$\left\{\begin{array}{l} {}^{C}\Delta_{a}^{\upsilon }e_{1}(t)=\sin(\pi y_{2}(\omega ))-x_{2}(\omega )-\gamma \delta x_{1}(\omega )+\delta y_{1}(\omega )x_{1}(\omega )+u_{1}(\omega ),\\ {}^{C}\Delta_{a}^{\upsilon }e_{2}(t)=c(1-x_{2}{}^{2}(\omega ))y_{2}(\omega )-y_{2}(\omega )+\delta (-y_{1}(\omega )+x_{1}{}^{2}(\omega ))+u_{2}(\omega ). \end{array}\right.$$

By substituting the Controller (17) into (18), the error dynamical system can be simplified as the following form:

(20) $$\left\{\begin{array}{l} {}^{C}\Delta_{a}^{\upsilon }e_{1}(t)=-e_{1}(\omega ),\\ {}^{C}\Delta_{a}^{\upsilon }e_{2}(t)=-e_{2}(\omega ). \end{array}\right.$$

For convenience of analysis, (20) is rewritten in the compact form

(21) $${}^{C}\Delta_{a}^{\upsilon }\left(e_{1}(t),e_{2}(t)\right)=N\times {\left(e_{1}(\omega ),e_{2}(\omega )\right)}^{T},$$

where $N=\left[\begin{array}{cc} -1 & 0\\ 0 & -1 \end{array}\right]$. It is clear that the eigenvalues of the matrix N satisfy the following stability condition:

(22) $$\left|\lambda_{i}\right|<{\left(2\cos\frac{\left|\arg\lambda_{i}\right|-\pi }{2-\upsilon }\right)}^{\upsilon }\;\mathrm{and}\;\left|\arg\lambda_{i}\right|>\frac{\upsilon \pi }{2},i=1,2$$

Therefore, the zero equilibrium point of (20) is globally, asymptotically stable according to Theorem 1, which implies that the hybrid synchronization between Maps (16) and (17) is realized. □

**Figure 9:**
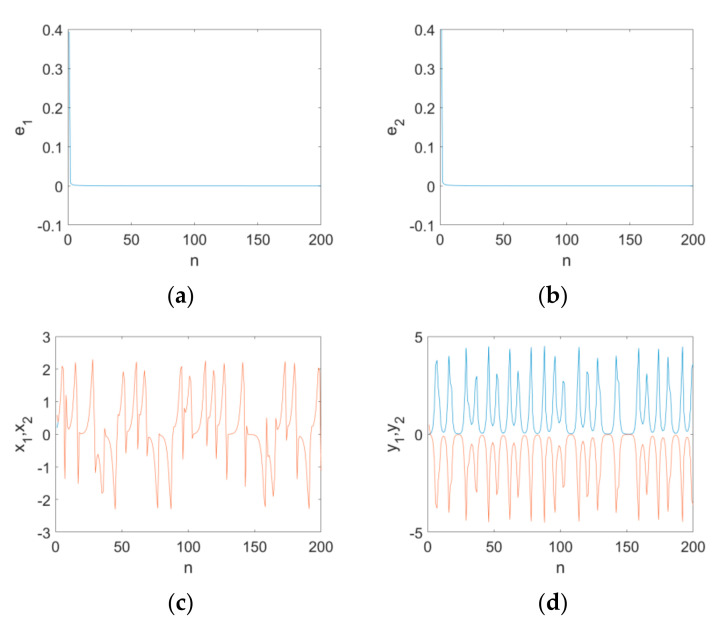
The simulation results for the synchronization of the map when υ=0.98 with the variation in n: (**a**) the error state variable $e_{1}$; (**b**) the error state variable $e_{2}$; (**c**) the state variables $x_{1},x_{2}$; (**d**) the state variables $y_{1},y_{2}$.

The numerical simulation results are depicted in Figure 9. The system parameters of the two maps are fixed as γ=1.25,δ=0.75,b=2.2,c=0.95, and the order is υ=0.98 in the numerical simulation. The initial conditions of (16) and (17) are $\left(x_{10},y_{10})=(0.1,0.1\right)$ and $\left(x_{20},y_{20})=(0.2,0.2\right)$, respectively. It can be seen that the error state variables $e_{1}$ and $e_{2}$ converge to zero rapidly as n increases (Figure 9a,b). In Figure 9c,d, the corresponding state variables of the maps are synchronized under the Controllers (18).

**Figure 10:**
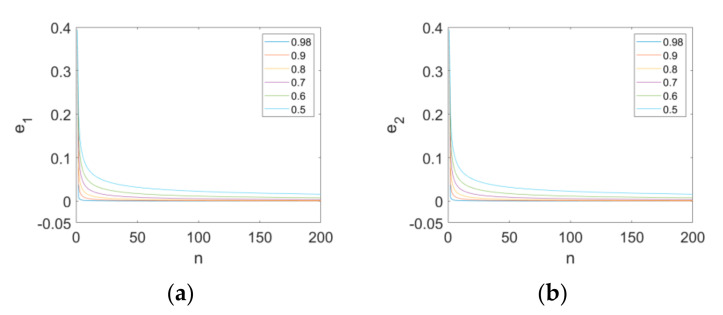
The error state variables of synchronization with different values of order: (**a**) the error state variable $e_{1}$; (**b**) the error state variable $e_{2}$.

Furthermore, the synchronization of Maps (16) and (17) with the same controllers and different orders (υ=0.98,0.9,0.8,0.7,0.6,0.5) is also analyzed, evident in Figure 10. Comparing the results in the case of υ=0.98, we find that the error state variables $e_{1}$ and $e_{2}$ need more time to converge to zero when the order decreases from 0.98 too. It can be concluded that the increase in the order will promote the synchronization speed of the Map (10).

## 6. Discussion

A novel fractional-order discrete map with a sinusoidal function is presented. Typical nonlinear features, including chaos and bifurcations, of the map are analyzed. The basic properties involving the stability of the equilibrium points and symmetry of the map are studied by theoretical analysis. The dynamics of the map in commensurate-order and incommensurate-order cases are investigated. The bifurcation types and influential parameters for the map are analyzed via bifurcation diagrams and maximum Lyapunov exponent spectrums. Hopf, period-doubling, and symmetry-breaking bifurcations are observed when a system parameter is varied. The bifurcation diagrams and maximum Lyapunov exponent spectrums, with both a variation in a system parameter and a derivative order or two orders, are shown in a three-dimensional space. The results indicate that the variation in the order has no effect on the symmetry-breaking bifurcation point. The heterogeneous hybrid synchronization of the map is realized by designing suitable controllers. Numerical simulations are carried out to verify the effectiveness of the controllers.

It is worth noting that an order of a fractional-order system is a very important parameter. For the map studied in the paper, the increase in the derivative order has no effect on the symmetry-breaking bifurcation point but can promote the synchronization speed. These results are important for the application of the fractional-order discrete sinusoidal map in encryption and secure communication. This is due to the fact that the rich dynamics of the map will increase the security of transmission signals. It lays a good foundation for the future analysis or engineering application of the fractional-order discrete map.

The influence of the order on the symmetry-breaking bifurcation point and synchronization speed is proved by the results of the fractional-order discrete sinusoidal map. However, its generalization for all the fractional-order maps is pending further research. The main reasons are the complex forms and rich dynamics of fractional-order discrete maps. Therefore, to establish the universality of the conclusion is one of our next aims. Furthermore, it is well known that global dynamics can obtain the main characteristics of a system from a global perspective and it is very important for practical application. Further work will consider the analysis of global dynamics for a fractional-order discrete map and try to apply it to synchronization control.

## Data Availability

The datasets generated and analyzed during the current study are available from the corresponding author on reasonable request.
